# Estimation of Spacecraft Angular Velocity Based on the Optical Flow of Star Images Using an Optimized Kalman Filter

**DOI:** 10.3390/biomimetics9120748

**Published:** 2024-12-09

**Authors:** Jiaqian Si, Yanxiong Niu, Haisha Niu, Zixuan Liu, Danni Liu

**Affiliations:** 1 School of Instrument Science and Opto-Electronics Engineering, Beihang University, Beijing 100191, China; sijiaqianbuaa@163.com (J.S.); niuyx@buaa.edu.cn (Y.N.); lzx961127@163.com (Z.L.); 2 School of Instrument Science and Opto-Electronics Engineering, Beijing Information Science and Technology University, Beijing 100192, China; niuhs@buaa.edu.cn; 3 College of Artificial Intelligence, China University of Petroleum, Beijing 100100, China

**Keywords:** biomimetic vision, angular velocity, Kalman filter, optical flow, star sensor

## Abstract

Biomimetic vision is a promising method for efficient navigation and perception, showing great potential in modern navigation systems. Optical flow information, which comes from changes in an image on an organism’s retina as it moves relative to objects, is crucial in this process. Similarly, the star sensor is a critical component to obtain the optical flow for attitude measurement using sequences of star images. Accurate information on angular velocity obtained from star sensors could guarantee the proper functioning of spacecraft in complex environments. In this study, an optimized Kalman filtering method based on the optical flow of star images for spacecraft angular velocity estimation is proposed. The optimized Kalman filtering method introduces an adaptive factor to enhance the adaptability under dynamic conditions and improve the accuracy of angular velocity estimation. This method only requires optical flow from two consecutive star images. In simulation experiments, the proposed method has been compared with the classic Kalman filtering method. The results demonstrate the high precision and robust performance of the proposed method.

## 1. Introduction

Satellites play a crucial role in deep space exploration, national defense infrastructure, and industrial production. Small satellites serve as a representative category within the satellite domain for the advantages of low weight, compact size, and cost-effectiveness. However, small satellites are constrained by limitations in mass, power consumption, and volume [1,2]. The successful execution of small satellite missions relies on the performance of onboard instruments and the precision of satellite attitude control. Therefore, a high-performance attitude control system is an essential prerequisite for ensuring their efficient operation [3].

As the attitude change of a system could be intuitively described by angular velocity, it is an essential component of the required information for satellite attitude control [4]. Satellite attitude sensors include sun sensors, magnetometers, star sensors, infrared earth sensors, gyroscopes, and so on. In previous study, gyroscopes were commonly used to estimate the angular velocity of spacecraft [5,6,7]. However, the reliability and precision of gyroscopes deteriorate during long-running processes, resulting in significant signal drift and noise [8,9].

**Figure 1:**
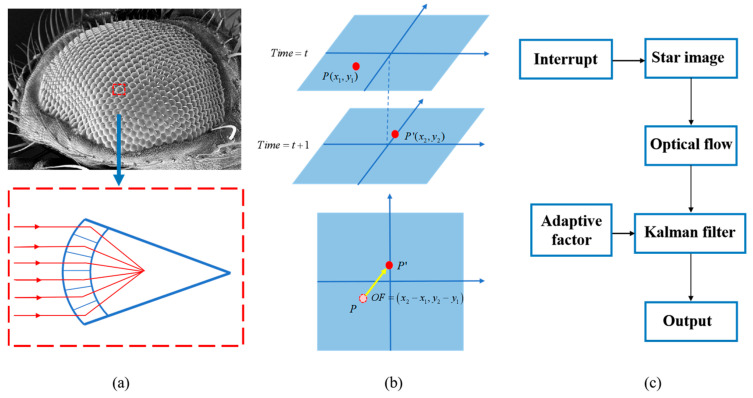
Bio-inspired optical-flow-based motion estimation and navigation system. (**a**) Compound eye structure and imaging modeling. (**b**) Optical flow calculation based on point displacement. (**c**) System workflow of proposed method for bio-inspired navigation.

In recent years, star sensors have been increasingly utilized in angular velocity estimation due to the associated high precision, ability to conceal, and absence of drift [10,11]. Drawing inspiration from the structure of an insect’s compound eye (Figure 1a), this approach mimics the ability to capture motion through an optical system. With advancements in CMOS technology, star sensors have become more power-efficient and miniaturized, making them highly suitable for small satellite applications. The integration of such bio-inspired mechanisms and star sensor data provides a lightweight, drift-free, and precise solution for attitude measurement in space navigation [12,13,14].

Estimation algorithms are researched for angular velocity estimation based on star sensors due to the noise associated with measurements [15]. In previous studies, two methods for estimating angular velocity have been identified: the derivation method and the estimation method. In the derivation method, angular velocity is directly obtained from the derivative of attitude. For example, the angular velocity information of the object can be derived by kinematically analyzing the attitude quaternion and its derivative. However, the accuracy of the angular velocity estimation is affected by the attitude measurement accuracy, as the attitude quaternion needs to be derived from the object’s kinematic model. In addition, measurement error is unavoidable in the measurements, which can lead to high-frequency noise when the derivative is taken. In the estimation method, angular velocity is obtained by state estimation based on a dynamic model. This method performs well. Furthermore, the problem associated with the time delay in the derivation method can be resolved in the estimation method [13,16,17,18]. Currently, the Kalman filtering method is one of the most representative estimation methods, which can be divided into linear and nonlinear Kalman filter algorithms [9]. Due to the limitations in equipment and computational capacity on small satellites, the linear Kalman filter is chosen as the preferred method in this study. The lower computational complexity and acceptable estimation accuracy meets the real-time requirements of the satellites system. Moreover, the uniform complete observability ensures that all system states can be inferred from observations over a finite time interval. In this way, the convergence of the Kalman filter is guaranteed [19,20,21,22].

The insect compound eye system can precisely detect the relative motion of surrounding objects by sensing the optical flow field within the view. Inspired by biological systems, biomimetic optical flow navigation technology enhances both the performance and the reliability of spacecraft attitude control [23,24,25,26,27]. By tracking the displacement of points in real-time star images, optical flow principles are used to compute angular velocity (Figure 1b). The star motion estimation is achieved by the optical flow method through grayscale information in two-dimensional coordinates. Compared with the star vector method using three-dimensional coordinates, the optical flow method achieves higher computational efficiency. Pal proposed an optical flow method based on the Kalman filter. This method is verified at constant slew velocities in simulation [13]. In practice, the motion of spacecraft often exhibits intricate dynamics and encompasses multiple movement modes. Ning conducted simulations in multiple scenarios involving diverse types of motion. But ultimate accuracy is highly dependent on measurement accuracy [28]. Lee and Kim proposed an angular velocity estimation method with a high sample frequency event camera [29,30]. However, the augmentation of the sampling frequency results in an escalated demand for data processing and storage. Excessive motion speed also leads to a decrease in star image quality, resulting in reduced estimation accuracy. Kun introduces a novel multiple-step approximate linear solution for estimating spacecraft angular velocity and the direction of the ground velocity vector using optical flow, which has poor accuracy of angular velocity [31]. Thus, they further address optical-flow-based angular velocity estimation [32]. However, the adaptability of this method is limited, and its usage is constrained by specific scenarios. In a word, the angular velocity estimation accuracy and adaptability of the algorithm is inadequate. Further studies are needed on optical flow for reliable angular velocity estimation.

In this study, we propose a novel and efficient optimized Kalman filtering method for estimating angular velocity based on optical flow, which incorporates information related to object velocity, orientation, and trajectory obtained from star image sequences. The system workflow (Figure 1c) incorporates star image acquisition, optical flow computation, and Kalman filter, dynamically refining the angular velocity estimation with an adaptive factor. In order to overcome the limitation of traditional Kalman filter, we have applied an adaptive Kalman filtering estimation method, and the resultant algorithm exhibits enhanced dynamic and adaptive performance. Four different trajectories with different dynamic conditions have been simulated. The angular velocity estimation results demonstrate that this method achieves high-precision angular velocity estimation under different dynamic conditions. The accuracy of angular velocity estimation is ca. 10^−5^ rad/s. The main contributions are shown as follows:

- The method utilized optical flow method for angular velocity estimation of the small satellites, reducing reliance on the precision of center-of-mass extraction in traditional star vector methods.
- The optimized Kalman filtering method incorporates an adaptive factor to enhance performance under dynamic conditions. It accommodates the modified Rayleigh distribution of acceleration with a non-zero mean, thereby improving angular velocity estimation to more accurately represent target maneuvering behavior.

## 2. Model for Optical Flow

Optical flow is commonly applied to measure the angular velocity of spacecraft in star sensors. Optical flow is a two-dimensional velocity field that describes motion of objects as an instantaneous, pixel-level velocity on the image plane [33,34,35].

The standard optical flow equation is derived under the assumption of gray invariance. Assuming that the gray value of a pixel is unchanged with object motion, the relationship between two continuous images is [36,37]:

(1) $$I\left(x,y,t\right)=I\left(x+dx,y+dy,t+dt\right)$$

where (*x*, *y*) are pixel coordinates. *I*(*x*, *y*, *t*) and *I*(*x + dx*, *y + dy*, *t + dt*) represent gray values of images at *t* and *t* + *dt*, respectively.

The Taylor series expansion is applied to the gray value expression at *t + dt*.

(2) $$I\left(x+dx,y+dy,t+dt\right)=I\left(x,y,t\right)+\frac{𝜕I}{𝜕x}\frac{dx}{dt}+\frac{𝜕I}{𝜕y}\frac{dy}{dt}+\frac{𝜕I}{𝜕t}+\varepsilon$$

where *ε* is a higher-order infinitesimal.

According to Equations (1) and (2):

(3) $$\frac{𝜕I}{𝜕x}\frac{dx}{dt}+\frac{𝜕I}{𝜕y}\frac{dy}{dt}+\frac{𝜕I}{𝜕t}=0$$

where ∂*I*/∂*x* and ∂*I*/∂*y* represent gray value gradients on the X-axis and Y-axis, respectively, denoted by *I_x_* and *I_y_*; *u* = *dx*/*dt* represents the velocity on the X-axis; *v* = *dy*/*dt* is the velocity on the Y-axis. The optical flow is described by the vector [*u v*]*^T^*. Thus, Equation (3) can be rewritten as:

(4) $$I_{x}u+I_{y}v+I_{t}=0$$

The grayscale change should be taken into consideration, leading to the change of formulation from Equation (4) to Equation (5), as follows:

(5) $$\left(I_{x}+\Delta I_{x}\right)u+\left(I_{y}+\Delta I_{y}\right)v+I_{t}=0$$

Equation (5) was decomposed and expressed as:

(6) $$I_{x}u+I_{y}v+I_{t}=-\left(\Delta I_{x}u+\Delta I_{y}v\right)$$

The temporal variation of gray level can be quantified as:

(7) $$\Delta I_{t}=\Delta I_{x}u+\Delta I_{y}v$$

## 3. Optical Flow Measurement Model of the Star Sensor

**Figure 2:**
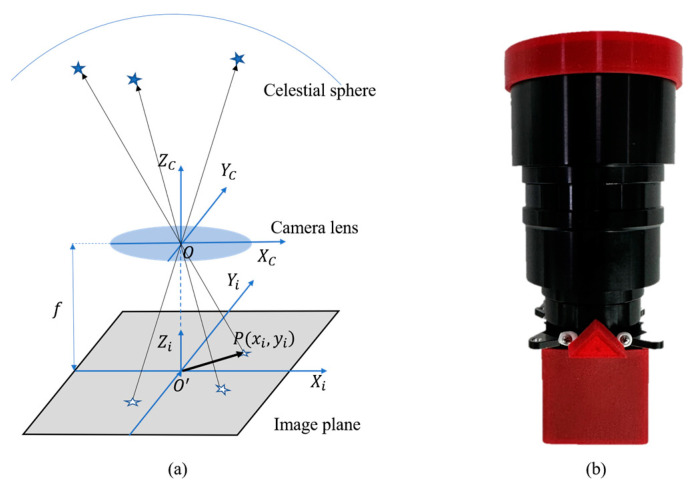
Star sensor imaging diagrams: (**a**) system imaging diagram; (**b**) image of the physical star sensor.

A star sensor is a device utilized for estimating spacecraft attitude through positional changes of navigation stars. Starlight is focused on the imaging plane through the lens of a star sensor [38]. The simplified diagram in Figure 2a, represented by the primary ray, illustrates the process of system imaging. Figure 2b is a physical image of the star sensor. The coordinate system associated with the camera lens is typically denoted as the camera coordinate system. It is a 3D coordinate system with the origin, $O_{c}$, located at the optical center of the camera lens, and the axes are defined relative to the orientation of the lens. The image plane coordinate system describes the position of stars on the 2D image plane. It is derived from the projection of 3D points in the camera’s coordinate system onto the 2D image plane, which lies perpendicular to the $Z_{c}$-axis. The coordinate origin $O_{i}$ is defined as the intersection point between the optical axis and the image plane. $\left(x_{c},y_{c},z_{c}\right)$ are the 3D coordinates of a point in the camera’s coordinate system. $P(x_{i},y_{i})$ is the corresponding point of a star on the image plane. f is the focal length of the star sensor.

Considering the infinite distance between navigation stars on the celestial sphere and star sensor, star imaging is regarded as the convergence of parallel rays from infinite distance. The relationship of star vector coordinates between the camera and image coordinate frames is described by Equations (8) and (9).

(8) $$x_{i}=-f\frac{x_{c}}{z_{c}}$$

(9) $$y_{i}=-f\frac{y_{c}}{z_{c}}$$

The location of *P* changes on the image plane with the motion of spacecraft [28]. *x* and *y* are obtained from location changes of navigation stars in two successive images at *t* and *t* − Δ*t*. The optical flows *u* and *v* are obtained by differentiating coordinates provided by Equations (8) and (9). *u* and *v* are as follows:

(10) $$u={\dot{x}}_{i}=-f\left(\frac{{\dot{x}}_{c}}{z_{c}}-\frac{x_{c}{\dot{z}}_{c}}{z_{c}^{2}}\right)$$

(11) $$v={\dot{y}}_{i}=-f\left(\frac{{\dot{y}}_{c}}{z_{c}}-\frac{y_{c}{\dot{z}}_{c}}{z_{c}^{2}}\right)$$

The parameter *u* represents the velocity vector in the *x* direction in Equation (10), and *v* is the velocity vector in the *y* direction in Equation (11).

The location of stars is unchanged relative to the inertial frame. The relationship between the star vector and the motion of spacecraft measured in the camera frame is as follows:

(12) $$\dot{N}\left(t\right)=-\omega \left(t\right)\times N\left(t\right)$$

where ***ω***(t) = [*ω_x_*(t) *ω_y_*(t) *ω_z_*(t)]*^T^* is the angular velocity in the camera frame. **N**(*t*) = (*x_c_*, *y_c_*, *z_c_*) is the star vector in camera frame.

By substituting Equation (12) into Equations (10) and (11), *u* and *v* are:

(13) $$u=f\left[\omega_{x}\frac{-x_{i}y_{i}}{f^{2}}+\omega_{y}(1+\frac{x_{i}^{2}}{f^{2}})+\omega_{z}\frac{y_{i}}{f}\right]$$

(14) $$v=f\left[-\omega_{x}(1+\frac{y_{i}^{2}}{f^{2}})+\omega_{y}\frac{x_{i}y_{i}}{f^{2}}-\omega_{z}\frac{x_{i}}{f}\right]$$

The discretization of *u* and *v* in Equations (13) and (14) at time instant *t* leads to the formulation of Equation (15):

(15) $$\left[\begin{array}{l} u\\ v \end{array}\right]=M\left.\left(x,\left.y\right)\right.\right|t\cdot \left[\begin{array}{l} \omega_{x}\\ {\dot{\omega }}_{x}\\ \omega_{y}\\ {\dot{\omega }}_{y}\\ \omega_{z}\\ {\dot{\omega }}_{z} \end{array}\right]$$

where

(16) $$M\left.\left(x,\left.y\right)\right.\right|t=\left[\begin{array}{cccccc} -\frac{x_{i}\left(t\right)y_{i}\left(t\right)}{f} & 0 & f+\frac{x_{i}^{2}\left(t\right)}{f} & 0 & y_{i}\left(t\right) & 0\\ -\left(f+\frac{y_{i}^{2}\left(t\right)}{f}\right) & 0 & \frac{x_{i}\left(t\right)y_{i}\left(t\right)}{f} & 0 & -x_{i}\left(t\right) & 0 \end{array}\right]$$

Angular velocity information can then be calculated by Equations (15) and (16) by the optical flow information [*u v*]*^T^* of the star.

## 4. Error Analysis of Optical Flow Estimation Based on Angular Velocity

In angular velocity estimation, the traditional optical flow equation introduces errors when the angular velocity changes, as the derivative of angular velocity is non-zero. This error primarily arises from the inability of the optical flow method to accurately reflect dynamic changes in angular velocity.

As the angular velocity ω varies with time *t*, the speed component of pixels within the image correspondingly adjusts. The true velocity vector representing pixel motion can be articulated as follows:

(17) $$u=\left|\omega \left(t\right)\right|\cdot r\cdot \cos\left(\theta \right)$$

(18) $$v=\left|\omega \left(t\right)\right|\cdot r\cdot \sin\left(\theta \right)$$

where *r* denotes the distance from the object to the center of rotation; *θ* represents the angle formed between vector $\omega \left(t\right)$ and the X-axis.

The assumption of grayscale invariance is based on the ideal scenario where the grayscale values of an object remain unchanged in adjacent frames. However, practical application scenarios are subject to various factors. The grayscale values of pixels may be influenced by the variations in lighting conditions and cumulative exposure time at a single pixel due to camera motion. Grayscale fluctuations might result in increased errors during optical flow estimation. In addition, the optical flow equation is based on a linear approximation using Taylor expansion. Under dynamic conditions, the neglect of these higher-order terms leads to substantial estimation errors.

The true angular velocity is represented as $\omega \left(t\right)$, while the estimated angular velocity derived from the optical flow equation is denoted as $\hat{\omega }\left(t\right)$. Consequently, the error can be defined as follows:

(19) $$\Delta \omega \left(t\right)=\hat{\omega }\left(t\right)-\omega \left(t\right)$$

Suppose the angular velocity at time $t_{0}$ is $\omega_{0}$, and its angular acceleration is $\dot{\omega }\left(t\right)$. Angular velocity at time $t_{0}+\Delta t$ is presented as:

(20) $$\omega \left(t\right)=\omega_{0}+\dot{\omega }(t)\cdot \Delta t$$

In the absence of prior information, when calculating optical flow solely based on the optical flow equation, the error $\Delta \omega \left(t\right)$ is:

(21) $$\Delta \omega \left(t\right)=-\dot{\omega }(t)\cdot \Delta t$$

The error $\Delta \omega \left(t\right)$ is directly proportional to the angular acceleration $\dot{\omega }\left(t\right)$. This error tends to accumulate over time. According to Equation (21), the error of optical flow can be described as:

(22) $$\Delta u=\left|\Delta \omega \left(t\right)\right|\cdot r\cdot \cos\left(\theta \right)$$

(23) $$\Delta v=\left|\Delta \omega \left(t\right)\right|\cdot r\cdot \sin\left(\theta \right)$$

To reduce the errors induced by variations in angular velocity, the optimized Kalman filtering method offers an effective solution. By combining the system’s dynamic model with observational data, the Kalman filter introduces prior information and updates the estimate, achieving the real-time correction of dynamic errors. This method ensures that the system maintains high accuracy and stability under various operating conditions.

## 5. Angular Velocity Estimation Using an Optimized Kalman Filter

In angular velocity estimation, analytical methods based on optical flow equations can effectively calculate the object motion. However, in practical applications with variable angular velocity, traditional optical flow equation introduces error due to the non-zero derivative of angular velocity. This error primarily arises from the insensitivity to the instantaneous changes in the object’s motion state. It leads to significant discrepancies between the estimated and true values in scenarios involving rapid rotation or varying speeds. To address this challenge, the use of an optimized Kalman filter has proven to be an effective solution. The Kalman filtering method is a recursive algorithm that dynamically adjusts estimation errors through combining system models with observational data. In angular velocity estimation, the Kalman filter continuously updates state information in real-time to better adapt to environmental changes and the dynamic characteristics of the system [39,40].

### 5.1. Prediction Model

Driven by white noise ***W***(*t*) and system control matrix ***U***(*t*), the prediction of the state is:

(24) $$\hat{X}(t,t-\Delta t)=\varphi (t,t-\Delta t)\hat{X}(t-\Delta t)+U\left(t\right)+W\left(t\right)$$

***X***(*t*) is the state vector. $\hat{X}(t,t-\Delta t)$ is the predicted state vector. φ(t,t−Δt) denotes the state transition matrix. *τ* is the time constant of the first-order Markov process. Equation (24) represents the prediction of angular velocity within a dynamic system.

Where

$$X\left(t\right)=\left[\omega_{x}\right.\left(t\right)\;{\dot{\omega }}_{x}\left(t\right)\;\omega_{y}\left(t\right)\;{\dot{\omega }}_{y}\left(t\right)\;{\left.\omega_{z}\left(t\right)\;{\dot{\omega }}_{z}\left(t\right)\right]}^{T}\in R^{6\times 1}$$

$$\begin{array}{c} \varphi (t,t-\Delta t)=\left[\begin{array}{ccc} \varphi_{x}(t,t-\Delta t) & 0 & 0\\ 0 & \varphi_{y}(t,t-\Delta t) & 0\\ 0 & 0 & \varphi_{z}(t,t-\Delta t) \end{array}\right]\\ \varphi_{x}(t,t-\Delta t)=\left[\begin{array}{cc} 1 & \tau_{x}\left(1-e^{-\Delta t/\tau_{x}}\right)\\ 0 & e^{-\Delta t/\tau_{x}} \end{array}\right]\\ \varphi_{y}(t,t-\Delta t)=\left[\begin{array}{cc} 1 & \tau_{y}\left(1-e^{-\Delta t/\tau_{y}}\right)\\ 0 & e^{-\Delta t/\tau_{y}} \end{array}\right]\;\\ \varphi_{z}(t,t-\Delta t)=\left[\begin{array}{cc} 1 & \tau_{z}\left(1-e^{-\Delta t/\tau_{z}}\right)\\ 0 & e^{-\Delta t/\tau_{z}} \end{array}\right]\; \end{array}$$

The covariance matrix of process noise $Q\left(t\right)$ is given by:

(25) $$Q\left(t\right)=\left(\begin{array}{ccc} \frac{2\sigma_{x}^{2}}{\tau_{x}}Q_{x}\left(t\right) & 0 & 0\\ 0 & \frac{2\sigma_{y}^{2}}{\tau_{y}}Q_{y}\left(t\right) & 0\\ 0 & 0 & \frac{2\sigma_{z}^{2}}{\tau_{z}}Q_{z}\left(t\right) \end{array}\right)$$

where

(26) $$Q_{x}\left(t\right)=Q_{y}\left(t\right)=Q_{z}\left(t\right)=\left(\begin{array}{cc} q_{11} & q_{12}\\ q_{21} & q_{22} \end{array}\right)\approx \left[\begin{array}{cc} \frac{\Delta t^{3}}{3} & \frac{\Delta t^{2}}{2}\\ \frac{\Delta t^{2}}{2} & \Delta t \end{array}\right]$$

σ^2^ represents the corresponding variance.

### 5.2. Measurement Model

Based on the dynamic equation, the optical flow measurements for the *k* stars are available. The measurement equation is:

(27) $$Z\left(t\right)=H\left(t\right)X\left(t\right)+V\left(t\right)$$

where

(28) $$\begin{array}{c} Z\left(t\right)={\left[z_{1}\left(t\right)\;z_{2}\left(t\right)\cdots z_{k}\left(t\right)\right]}^{T}\\ ={\left[u_{1}\left(t\right)\;v_{1}\left(t\right)\;u_{2}\left(t\right)\;v_{2}\left(t\right)\;\cdots \;u_{k}\left(t\right)\;v_{k}\left(t\right)\right]}^{T}\\ H\left(t\right)=\left[\begin{array}{l} h_{1}\left(t\right)\\ h_{2}\left(t\right)\\ \vdots \\ h_{k}\left(t\right) \end{array}\right]=\left[\begin{array}{c} M_{1}\left(x,y\right)\left|t\right.\\ M_{2}\left(x,y\right)\left|t\right.\\ \vdots \\ M_{k}\left(x,y\right)\left|t\right. \end{array}\right] \end{array}$$

***Z***(t) is the observation vector at time *t*. **H**(t) is the observation matrix that maps the state to the measurement space. ***V***(*t*) represents a white Gaussian noise process, and the statistical character of the process follows ***V***(*t*)~***N*** (0, ***R***(*t*)), where

(29) $$R_{k}\left(t\right)=\left[\begin{array}{ll} \sigma_{1}^{2} & 0\\ 0 & \sigma_{2}^{2} \end{array}\right]$$

The initial values of the Kalman filter are determined based on the system’s prior knowledge and the measurement information from the sensors. Finally, the updated state of the stars is obtained from:

(30) $$\left\{\begin{array}{l} K_{k}\left(t\right)=P_{k/k-1}\left(t\right)h_{k}^{T}\left(t\right){\left(h_{k}\left(t\right)P_{k/k-1}\left(t\right)h_{k}^{T}\left(t\right)+R_{k}\left(t\right)\right)}^{-1}\\ {\hat{X}}_{k}\left(t\right)={\hat{X}}_{k-1}\left(t\right)+K_{k}\left(t\right)\left(z_{k}\left(t\right)-h_{k}\left(t\right){\hat{X}}_{k-1}\left(t\right)\right)\\ P_{k}\left(t\right)=\left(I-K_{k}\left(t\right)h_{k}\left(t\right)\right)P_{k/k-1}\left(t\right) \end{array}\right.$$

where **K**(*t*) is the filtering gain. **P**_*k*/*k*−1_ represents the predicted state error covariance. The scale of the inverse matrix is reduced using this method, which saves computing time and storage space.

### 5.3. Adaptive Model

To ensure superior tracking performance, it is essential to thoroughly investigate the motion of the targets. The current statistical model is capable of estimating the mean value of the instantaneous acceleration of the target state while simultaneously providing real-time updates.

In the current statistical model, a constant value is assigned to the variance of maximum angular acceleration. The variance is expressed as:

(31) $$\left\{\begin{array}{l} \sigma_{x}^{2}=\frac{4-\pi }{\pi }{\left[a_{x\max}-a_{x}\right]}^{2}\;\;\;\;\;\;\;\;a_{x}\geq 0\\ \sigma_{x}^{2}=\frac{4-\pi }{\pi }{\left[a_{x}-a_{-x\max}\right]}^{2}\;\;\;\;\;\;a_{x}<0 \end{array}\right.$$

Similar expressions are applicable for the *y* and *z* directions. *a_xmax_* and *a*_−*xmax*_ represent the maximum angular accelerations in positive and negative *x* direction. In Equation (31), the acceleration noise variance of the modified Rayleigh distribution is determined by the estimated acceleration at each step.

The current statistical model leverages the present estimation to adapt the system parameters. It achieves a more precise reflection of the maneuvering target’s current characteristics. However, the accuracy decreases when the acceleration is significantly lower than the maximum set value. The filter is invalid when the angular acceleration exceeds the maximum set value. To address this problem, an innovative approach was introduced, where

(32) $$G_{k}\left(t+\Delta t\right)=z_{k}\left(t+\Delta t\right)-h_{k}\left(t+\Delta t\right)\hat{X}\left(t+\Delta t,t\right)$$

where ***G****_k_*∈R^2×1^, and the covariance $S_{k}\left(t+\Delta t\right)$ is given by:

(33) $$S_{k}\left(t+\Delta t\right)=h_{k}\left(t+\Delta t\right)P\left(t+\Delta t,t\right)h_{k}^{T}\left(t+\Delta t\right)+R_{k}\left(t+\Delta t\right),S_{k}\in R^{2\times 2}$$

The adaptive factor is defined as:

(34) $$\alpha \left(t\right)=\exp\left(D\left(t\right)/\eta -1\right)$$

where ***D***(*t*) is defined as ***G****^T^*(*t)**S***^−1^(*t*)***G***(*t*). The tuning parameter is denoted as *η*. It is set to 0.36 based on the dynamic range of the spacecraft. Therefore, $\varphi_{x}\left(t,t-\Delta t\right)$ can be represented as:

(35) $$\varphi_{x}\left(t,t-\Delta t\right)=\left(\begin{array}{cc} 1 & \tau_{x}\left(1-e^{-\Delta t/\tau_{x}}\right)/\alpha \left(t\right)\\ 0 & e^{-\Delta t/\tau_{x}} \end{array}\right)$$

We can rewrite the maximum acceleration as:

(36) $$a_{\pm x\max}\left(t\right)=\alpha \left(t\right)a_{\pm x\max}$$

Similar expressions apply to the *y* and *z* directions. By incorporating an adaptive factor $\alpha \left(t\right)$, the current state is included in the present estimation. Therefore, a real-time acceleration update is facilitated, and the overall adaptability is enhanced.

## 6. Simulation

The star images are usually difficult to obtain due to access restrictions and limited availability. The simulated star images derived from actual astronomical catalogues provide an alternative approach. These simulations effectively replicated actual observations with high fidelity. The generated simulated star images were based on precise stellar catalogues to ensure the angular positions and magnitudes of the stars corresponded accurately with genuine celestial data. The star sensor field of view is 10° × 10°, with an image plane of 2048 × 2048 pixels and data sample frequency of 10 Hz. The star sensor is fixed on the spacecraft. Simplifying the measurement model, the spacecraft frame and the detector frame are assumed to be coincident in space. The simulation was performed using MatlabR2015b with the CPU at 2.5 GHz, a total work time of 300 s.

In the simulation algorithm, **H**_0_ is related to the position of star points. The other initial values of the Kalman filter are set as follows:

$$X_{0}={\left[0\;0\;0\;0\;0\;0\right]}^{T}$$

$$\phi_{0}=\left(\begin{array}{llllll} 1 & 1 & 0 & 0 & 0 & 0\\ 0 & 0 & 1 & 1 & 0 & 0\\ 0 & 0 & 0 & 0 & 1 & 1\\ 0 & 1 & 0 & 0 & 0 & 0\\ 0 & 0 & 0 & 1 & 0 & 0\\ 0 & 0 & 0 & 0 & 0 & 1 \end{array}\right)$$

$$P_{0}=\left(\begin{array}{cccccc} 0.09^{2} & 0 & 0 & 0 & 0 & 0\\ 0 & 0.009^{2} & 0 & 0 & 0 & 0\\ 0 & 0 & 0.09^{2} & 0 & 0 & 0\\ 0 & 0 & 0 & 0.009^{2} & 0 & 0\\ 0 & 0 & 0 & 0 & 0.09^{2} & 0\\ 0 & 0 & 0 & 0 & 0 & 0.009^{2} \end{array}\right)$$

$$Q_{0}=\left(\begin{array}{cc} 0.01 & 0\\ 0 & 0.1 \end{array}\right)$$

The spacecraft may undergo diverse forms of motion during operation. In order to ensure the authenticity and comprehensiveness of the simulation, the spacecraft trajectory was simulated in four dynamic conditions: a uni-axis fixed angular velocity motion, a uni-axis fixed angular acceleration motion, a uni-axis sinusoidal angular velocity motion, and a dual-axis fixed angular velocity motion. The simulation experiments were randomly repeated at the set velocities, and the root mean square error (RMSE) of angular velocity was calculated by Equation (37) in order to further assess the effectiveness of the method.

(37) $$\Delta \omega_{RMSE}=\sqrt{\frac{\sum_{n}^{l=1}{\left({\hat{\omega }}_{l}-\omega_{l}\right)}^{2}}{n}},\;l=1,2,3,\cdots ,n$$

**Figure 3:**
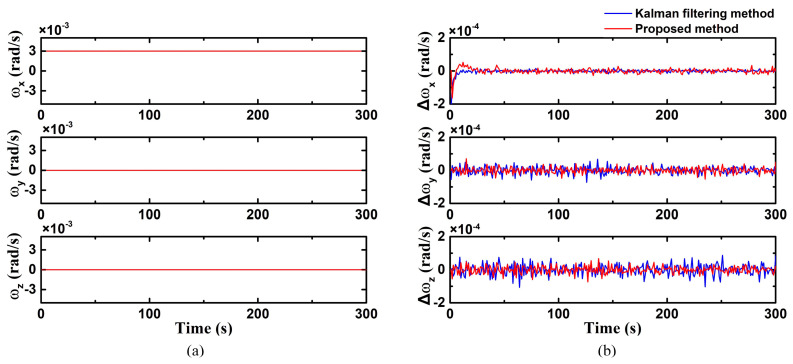
Uniaxial (X-axis) fixed angular velocity motion ((**a**) true value of triaxial angular velocity; (**b**) triaxial angular velocity error calculated by improved Kalman filter).

In case 1, the uniaxial (X-axis) fixed angular velocity motion was simulated, and the results are shown in Figure 3. In this case, the initial spacecraft angular velocity was 0. The spacecraft angular velocity undergoing temporal variations around these axes are shown in Figure 3a, where

(38) ω=[0.003 rad/s, 0 rad/s, 0 rad/s]

The angular velocity estimation errors of the two methods are illustrated in Figure 3b with different colors. It shows that both methods exhibit similar convergence speeds in the X-axis direction. The proposed method demonstrates an estimation error of 1.61 × 10^−5^ rad/s under a constant angular velocity, while the traditional Kalman filtering method displays an angular velocity estimation error at 1.65 × 10^−5^ rad/s. The results indicate that both methods achieve enhanced estimation performance under low-dynamic conditions. Moreover, the error in the Z-axis is comparatively larger due to the reduced sensitivity of the star sensor along the optical axis.

In case 2, the angular velocity on the X-axis is involved as uniform acceleration motion, where

(39) $$\omega =[\frac{0.00375}{T}t\;rad/s,\;0\;rad/s,\;0\;\mathrm{rad}/s]$$

**Figure 4:**
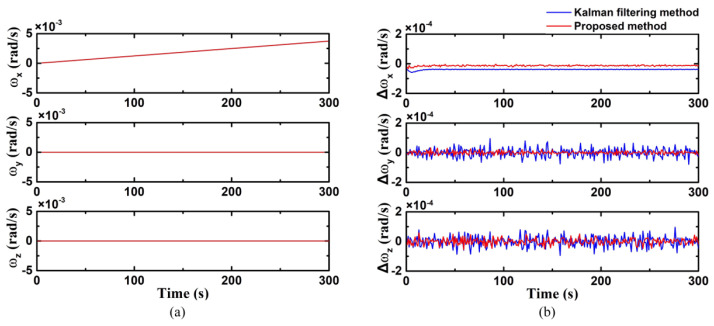
Uniaxial (X-axis) fixed angular acceleration motion ((**a**) true value of triaxial angular velocity; (**b**) triaxial angular velocity error calculated using the improved Kalman filter).

*T* is the total work time. Similarly, angular velocity of three axes is displayed in Figure 4a. And the estimation errors are shown in Figure 4b.

It clearly demonstrates that the error of the proposed method is significantly smaller than that of the Kalman filtering method. The error of the proposed method exhibits better performance as the angular velocity of the X-axis increases, approaching closer to zero. And the errors on the other two axes are also smaller. The estimation accuracy of the improved Kalman filtering method is 1.34 × 10^−5^ rad/s, and the calculation accuracy of the conventional Kalman filter is in the range of 3.88 × 10^−5^ rad/s.

In case 3, the angular velocity on the X-axis was a sine function. The triaxial angular velocity was:

(40) $$\omega =\left(0.01\sin\left(\frac{3\pi t}{100}\right)\;rad/s,0\;rad/s,0\;rad/s\right)$$

**Figure 5:**
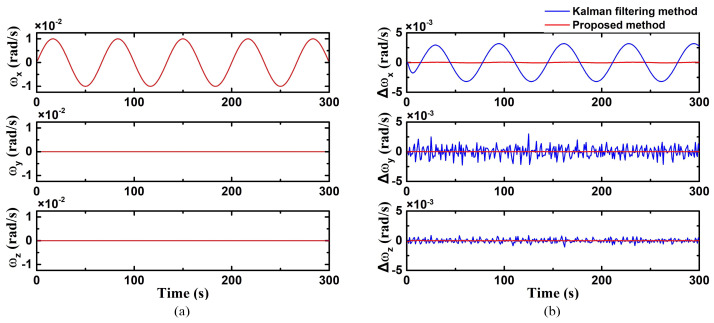
Uniaxial (X-axis) sinusoidal angular velocity motion ((**a**) true value of triaxial angular velocity; (**b**) triaxial angular velocity error using the improved Kalman filter).

As shown in Figure 5, the error on the X-axis of the proposed method is small and approaches 0. However, the conventional Kalman filtering method is susceptible to factors such as the magnitude and rate of change in angular velocity, resulting in significant fluctuations and demonstrating periodic variations in errors. The Kalman filtering method exhibits its maximum error at the peak, which is 3.2 × 10^−3^ rad/s. The RMSE of the improved Kalman filter on the X-axis is 3.88 × 10^−5^ rad/s, while the accuracy of the standard Kalman filtering method falls within 2.21 × 10^−3^ rad/s. The accuracy of the conventional Kalman filtering method decreased markedly under dynamic conditions, while the improved Kalman filtering method delivers superior dynamic performance.

**Figure 6:**
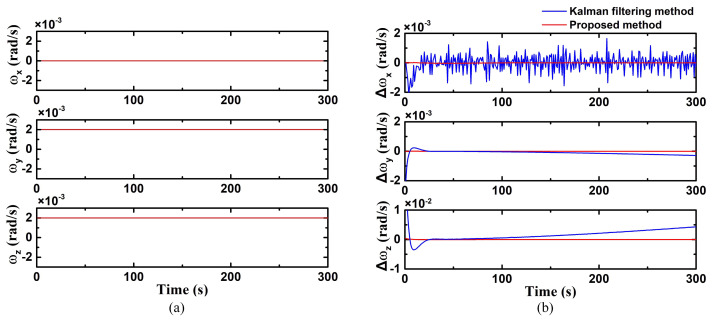
Dual-axis (Y- and Z-axis) fixed angular velocity motion ((**a**) true value of triaxial angular velocity; (**b**) triaxial angular velocity error using the improved Kalman filter).

In case 4, the situation involving a dual-axis was simulated. The angular velocities on the Y-axis and Z-axis are uniform motion. As demonstrated in Figure 6a, the angular velocities are set as:

(41) ω=[0 rad/s, 0.002 rad/s, 0.002 rad/s]

The estimation error of both methods of the dual-axis fixed-angle velocity are shown in Figure 6b. Compared with the proposed method, the conventional Kalman filtering method exhibits a longer convergence time. Moreover, there are also gradual increases in error on the Y-axis and Z-axis over time. The RMSEs of the conventional Kalman filtering method on Y-axis and Z-axis are 1.57 × 10^−3^ rad/s and 2.36 × 10^−3^ rad/s, while the RMSEs of the proposed method are 9.03 × 10^−6^ rad/s and 2.98 × 10^−5^ rad/s, respectively.

**Figure 7:**
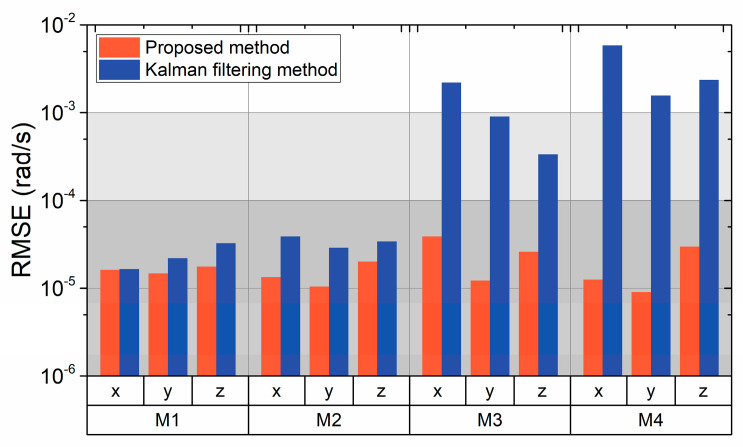
Estimated results and comparison.

Taking an overview of the four motions considered in the simulations, the errors associated with the improved Kalman filtering method and the conventional Kalman filtering method are presented in Figure 7. In the first and second cases, both the improved method and the traditional Kalman filtering method show an equivalent level of accuracy. In the third and fourth cases, the angular velocity error is greatly influenced by the dynamic conditions as the spacecraft motion is complex. Under these motions, the RMSEs exhibited by the conventional Kalman filtering method are significantly lower, and the accuracy of the proposed method is superior, with dynamic parameters adjusted in real time.

## 7. Conclusions

The optical flow method based on biomimetic vision is widely used in measuring the angular velocity of star sensors, applying the Kalman filtering method for iterative calculation. However, the accuracy of the conventional Kalman filtering method does not satisfy spacecraft requirements in highly dynamic situations. Due to the poor dynamic performance of these methods, it is difficult to achieve high-precision angular velocity estimation. In the working process of spacecraft, there are various motion patterns. It is necessary to enhance adaptability and robustness of the algorithm.

An optical flow method using an improved Kalman filter is proposed in this study. In our approach, the kinetic paraments are applied in the model, which ensures improved performance in terms of adaptability and stability. The improved current statistical filtering method is applied in this paper. The statistical characteristics of acceleration satisfy the modified Rayleigh distribution with a non-zero mean, which is in accordance with the actual maneuvering characteristics of maneuvering targets. The variance of the Rayleigh distribution is determined by the mean. While estimating the state of the target, the real-time mean of the maneuvering acceleration is obtained; thereby, the distribution of acceleration is corrected in real time. It is fed back into the filter gain of the next moment by variance, achieving closed-loop tracking with good adaptivity. Under high dynamic conditions, the accuracy of the proposed method is superior to the Kalman filtering method. Compared with conventional Kalman filter, the experimental results demonstrate enhanced accuracy of the proposed method and more robust performance in the four simulation cases.

## Data Availability

The data that support the findings of this study are available within the article and from the corresponding author upon reasonable request.
